# A newly identified IncY plasmid from multi-drug-resistant *Escherichia coli* isolated from dairy cattle feces in Poland

**DOI:** 10.1128/spectrum.00877-24

**Published:** 2024-07-16

**Authors:** Magdalena Zalewska, Aleksandra Błażejewska, Jan Gawor, Dorota Adamska, Krzysztof Goryca, Michał Szeląg, Patryk Kalinowski, Magdalena Popowska

**Affiliations:** 1 Department of Bacterial Physiology, Institute of Microbiology, Faculty of Biology, University of Warsaw, Warsaw, Poland; 2 DNA Sequencing and Synthesis Facility, Institute of Biochemistry and Biophysics, Polish Academy of Sciences, Warsaw, Poland; 3 Genomics Core Facility, Centre of New Technologies, University of Warsaw, Warsaw, Poland; Taichung Veterans General Hospital, Taichung, Taiwan

**Keywords:** antibiotic resistance, cattle manure, *Escherichia coli*, insertion elements, multi-drug resistance, plasmids

## Abstract

Comprehensive whole-genome sequencing was performed on two multi-drug-resistant *Escherichia coli* strains isolated from cattle manure from a typical dairy farm in Poland in 2020. The identified strains are resistant to beta-lactams, aminoglycosides, tetracyclines, trimethoprim/sulfamethoxazole, and fluoroquinolones. The complete sequences of the harbored plasmids revealed antibiotic-resistance genes located within many mobile genetic elements (e.g., insertional sequences or transposons) and genes facilitating conjugal transfer or promoting horizontal gene transfer. These plasmids are hitherto undescribed. Similar plasmids have been identified, but not in Poland. The identified plasmids carried resistance genes, including the tetracycline resistance gene *tet(A*), aph family aminoglycoside resistance genes *aph(3*″)*-lb* and *aph (6)-ld*, beta-lactam resistance genes *bla*_TEM-1_ and *bla_CTX-M-15_*, sulfonamide resistance gene *sul2*, fluoroquinolone resistance gene *qnrS1*, and the trimethoprim resistance gene *dfrA14*. The characterized resistance plasmids were categorized into the IncY incompatibility group, indicating a high possibility for dissemination among the *Enterobacteriaceae*. While similar plasmids (99% identity) have been found in environmental and clinical samples, none have been identified in farm animals. These findings are significant within the One Health framework, as they underline the potential for antimicrobial-resistant *E. coli* from livestock and food sources to be transmitted to humans and *vice versa*. It highlights the need for careful monitoring and strategies to limit the spread of antibiotic resistance in the One Health approach.

**IMPORTANCE:**

This study reveals the identification of new strains of antibiotic-resistant *Escherichia coli* in cattle manure from a dairy farm in Poland, offering critical insights into the spread of drug resistance. Through whole-genome sequencing, researchers discovered novel plasmids within these bacteria, which carry genes resistant to multiple antibiotics. These findings are particularly alarming, as these plasmids can transfer between different bacterial species, potentially escalating the spread of antibiotic resistance. This research underscores the vital connection between the health of humans, animals, and the environment, emphasizing the concept of One Health. It points to the critical need for global vigilance and strategies to curb the proliferation of antibiotic resistance. By showcasing the presence of these strains and their advanced resistance mechanisms, the study calls for enhanced surveillance and preventive actions in both agricultural practices and healthcare settings to address the imminent challenge of antibiotic-resistant bacteria.

## INTRODUCTION

*Escherichia coli* is a bacterial species found in the environment or colonizing the gastrointestinal tract of humans and animals. While most strains of *E. coli* are harmless commensals, some are pathogenic lineages capable of causing severe intestinal and extraintestinal diseases such as sepsis, urinary tract infections, or meningitis (1). In addition, very strong evidence of gene exchange between strains found in food and clinics has been recorded (2). *E. coli* represents a major reservoir of antibiotic resistance genes (ARGs) that may be responsible for therapeutic failures in both human and veterinary medicine. Their numbers in *E. coli* isolates have increased during recent decades, with many of these ARGs being acquired by horizontal gene transfer (HGT). In the enterobacterial gene pool, *E. coli* may act as a donor and recipient of ARG, i.e., acquiring ARGs, such as the aminoglycoside-resistance gene *armA*, from bacteria and passing them to others.

The HGT allows bacteria to adapt to unfavorable environmental conditions by acquiring sections of DNA; these genes can often include foreign DNA that can extend the ecological capabilities of the recipient. They can modify how the microorganism interacts with its host, give it an advantage over other microbes in the same environment, and, significantly, make it resistant to primary antibiotics by carrying ARGs on mobile genetic elements (MGEs). The roles of these MGEs are varied, and in addition to conferring antibiotic resistance, they can facilitate the digestion of various carbohydrate types, metal resistance, virulence, and catabolic processes beneficial for xenobiotic biodegradation in the environment (3). HGT is widespread and enables organisms from different taxonomic groups to share genetic material, effectively eroding the genetic distinctions between distinct phylogenetic groups and accounting for the substantial gene content variability among closely related prokaryotes (4).

A key role in HGT is played by plasmids. These allow genetic material to be transferred at high rates through several pathways, primarily conjugation (including plasmid mobilization and conduction), but also through transduction, transformation, and vesiduction. Plasmids exhibit remarkable diversity with regard to size, copy number, G + C content, replication mechanisms, modes of transmission, DNA structure (either circular or linear), genetic content, and host spectrum. They harbor a wide array of traits that bolster their persistence and broaden the environmental niches that their hosts can occupy. Plasmids contain essential genes for both vertical and horizontal transmission, including those for replication and conjugation, and may also encode specialized systems to enhance transmission and stability. Many plasmids possess “accessory” genes that enrich the host’s ecological niche by enabling the degradation of toxic substances or introducing novel metabolic pathways. Notably, plasmids with virulence genes and ARGs play pivotal roles in the unchecked proliferation of bacterial pathogens within the One Health continuum (5, 6).

For instance, in 2003, the *armA* gene was identified on a conjugative plasmid in *Klebsiella pneumoniae* (7). Since then, this gene has been found in several enterobacteria, including *Proteus mirabilis, Serratia marcescens, Acinetobacter baumannii*, and *Pseudomonas aeruginosa* (8–10). In 2005, *armA* was found in *E. coli* isolated from farm animals, and its prevalence is still increasing.

ESBL (extended-spectrum beta-lactamases)/AmpC genes have been found to be highly prevalent in commensal *E. coli* isolated from fecal samples of various food-producing animals, and many of them have been found to be located on plasmids. Several plasmid-encoded resistance mechanisms to quinolones have been identified in animal- and human-related sources, such as Qnr-like proteins, which protect DNA from binding to quinolone, the acetyltransferase that modifies certain fluoroquinolones, and active efflux pumps.

As a consequence of the high selective pressure imposed by the widespread use of tetracyclines, many bacteria, including *E. coli*, have developed or acquired MGE resistance to tetracycline: nine tetracycline efflux genes (*tetA, tetB, tetC, tetD, tetE, tetG, tetJ, tetL*, and *tetY)*, two tetracycline resistance genes encoding ribosomal protective proteins (*tetM* and *tetW)*, and one gene coding for an oxidoreductase that inactivates tetracyclines (*tetX*) have been identified in *E. coli*. The major mechanisms of tetracycline resistance encountered in *E. coli* of animal origin cover (i) the active efflux by proteins of the major facilitator superfamily and (ii) ribosome protection (11).

In addition, aminoglycoside resistance genes, which can be classified as target modification (*armA, rmtA,* and *nmpA*) or antimicrobial-inactivating (most prevalent *strA* and *strB*) enzymes, have been found in *E. coli* mobilized on resistance plasmids. These provide resistance against only the most extensively studied antimicrobial groups applied for both human and animal medicines; more mechanisms have been reviewed in detail by Poirel et al. (11).

Generally, ARGs in *E. coli* are considered a major global challenge in human and animal medicine and must be considered a real public health concern. *E. coli* infections in animals are treated with a range of antimicrobials; ampicillin, streptomycin, sulfonamides, or oxytetracyclines are commonly used to treat bovine mastitis and newborn diarrhea, with broad-spectrum cephalosporins and fluoroquinolones also possibly being used. Animal feces are important reservoirs of human pathogens such as pathogenic *E. coli*, *Salmonella,* or *Listeria* (12). The co-occurrence of various ARGs originating in animal intestines and microbial human pathogens, together with MGEs, creates perfect conditions for disseminating antibiotic resistance through the acquisition of ARGs by bacteria (13).

The present study examines *E. coli* strains extracted from the feces of dairy cattle, specifically from intensive breeding facilities. The antimicrobial susceptibility and plasmid profiles of the strains were tested, and the most common strains were selected for sequencing using the NovaSeq 6000 (Illumina) and MinIon (Oxford Nanopore) platforms. As a result, the study presents the full structure of the plasmids and the genetic information contained therein.

## MATERIALS AND METHODS

### Isolation of *E. coli* strains

Samples of dairy cattle manure were collected from a Polish dairy farm employing an intensive rearing system. The animals were highly productive Polish Holstein-Friesian dairy cows of the Black and White variety. The herd was located in the central part of Poland in the Masovian district. The manure sampling procedure is described in more detail by Zalewska et al. (14). To isolate antibiotic-resistant *E. coli* strains, the waste sample was enriched in Luria-Bertani broth by adding 1 g of feces to 9 mL of liquid medium. The cultures were incubated at 30°C and 37°C for 24 and 48 hours. After incubation, the bacterial suspensions were diluted (10^−1^, 10^−2^, and 10^−3^), and 100 µL of undiluted sample and each dilution were plated onto eosin methylene blue (EMB) agar and MacConkey agar: one set was plated onto the two media supplemented with imipenem (16 mg/L) to confirm a carbapenem resistance phenotype, and another set on the media supplemented with cefotaxime (4 mg/L) to test for an extended-spectrum beta-lactamase phenotype (15). Each isolation was performed as three biological replicates, followed by three technical ones. All selective media were purchased from Biomaxima (Poland). Antibiotics were purchased from Sigma-Aldrich (Merck, USA). The plates were incubated at 30°C and 37°C for 24 hours to select for animal pathogens and environmental strains. Next, approximately 24–48 colonies showing the morphology of the intended bacteria were taken and applied to three consecutive streaks to get pure colonies. The pure cultures were stored in PBS/glycerol stocks (20%, vol/vol) for further analysis.

### The susceptibility profiles of *E. coli* strains

The antibiotic susceptibility profiles of the isolated strains were determined by The Kirby–Bauer test (16). The phenotypic characterization was based on the following antimicrobial discs: imipenem (10 µg), ciprofloxacin (5 µg), cefotaxime (5 µg), and gentamicin (10 µg) (Oxoid, USA). The inhibition zones were measured according to EUCAST recommendations, i.e., after incubation for 18 hours at 37°C.

Strains with different antimicrobial susceptibility profiles, characterized by a difference in bacterial growth inhibition zone diameter of approximately ±2 mm around at least one antibiotic disk, were considered non-repetitive and chosen for further research. The isolated strains were confirmed as *E. coli* by MALDI-TOF MS/MS (matrix-assisted laser desorption/ionization system equipped with a time-of-flight mass spectrometer) (17). The analyses were performed in an external medical laboratory, ALAB Laboratoria Sp. z o. o. (Poland), according to a standard diagnostic procedure. Identification was performed by aligning the peaks to the best matching reference data, with the resulting log score classified as follows: ≥2.3 indicates a highly probable species; 2.0–2.3, a certain genus and probable species; 1.7–2.0, a probable genus; and <1.7, non-reliable identification. Antibiotic susceptibility testing was then performed with Vitek2 Compact equipment (BioMerieux, France) (18), with strains being classified as sensitive (S), resistant (R), or intermediate (I) based on the results. The AST-N331 susceptibility card was used. Strains with the same resistance profile were considered clones.

The plasmid profile of the non-repetitive strains was determined in three ways: (i) isolation of plasmid DNA with a commercially available kit—Plasmid Mini (A&A Biotechnology, Poland); (ii) rapid alkaline lysis for the isolation of plasmid DNA (19); (iii) plasmid visualization by Eckhardt electrophoresis (20). Examples of plasmid DNA with similar profiles were additionally differentiated by digestion with restriction enzymes BamHI and HindIII (NEB, USA). The digestion was performed according to the manufacturer’s recommendation. Depending on the product size, the isolation and digestion effect was visualized with electrophoresis in 1.5%–2% agarose. Strains with different profiles were chosen for sequencing.

### DNA extraction and sequencing

Genomic DNA was isolated from bacterial strains using Genomic Mini (A&A Biotechnology, Gdynia, Poland). The DNA concentration was measured using a Qubit fluorometer and a dsDNA High Sensitivity Assay Kit (Thermo Fisher Scientific, USA), and purity was determined by measuring the A260/A280 absorbance ratio with a NanoDrop spectrophotometer (Thermo Fisher, USA). Only samples with concentrations higher than 10 ng/µL and an A260/A280 ratio ranging from 1.8 to 2.0 were analyzed. DNA samples were stored at −20°C for further use. The DNA samples were isolated in triplicate.

### Genomic DNA sequencing on Oxford Nanopore MinION

Samples composed of 500 ng of isolated DNA were taken for library preparation. They were first mechanically fragmented using a syringe-based method (0.4 × 20 mm needle, 1 mL glass syringe) in a volume of 200 µL. The fragmented samples were purified and concentrated to a volume of 13 µL using the solid phase reversible immobilization method with Kapa Pure Beads (Roche, cat. no. 07983298001). Elution was performed for 10 minutes at 37°C. Libraries were constructed using the manufacturer’s protocol of Native Barcoding Kit 24 with genomic DNA (SQK-NBD112.24, version: NBE_9134_v112_revE_01Dec2021) using a NEB Blunt/TA Ligase Master Mix (New England Biolabs, cat. no. M0367), a NEBNext Quick Ligation Reaction Buffer (New England Biolabs, cat. no. B6058), and a NEBNext Companion Module for Oxford Nanopore Technologies (New England Biolabs, cat. no. E7180S). Sequencing was performed on the NanoPore MINIon MkB1 instrument using the R10.4 version of reagents (cat. no. FLO-MIN112) following the manufacturer’s protocol. Basecalling was performed with guppy (version 6.1.2) with --flowcell FLO-MIN112 --kit SQK-NBD112-24 options.

### Genomic DNA sequencing on Illumina NovaSeq 6000

The DNA samples were fragmented using the S220 Focused-ultrasonicator (Covaris) to a mean size of approximately 300 bp (Duty Factor: 10%, Peak Incident Power: 140, Cycles per Burst: 200, and Treatment Time: 80 s). Subsequently, libraries were constructed using the KAPA Hyper Prep Kit (Roche, cat. no. 07962363001) and TruSeq DNA UD Indexes adapters (Illumina, cat. no. 20020590) according to the manufacturer’s recommendations, starting with 100 ng of material. Five cycles of PCR amplification were performed for enrichment. Fragment size selection was carried out using Kapa HyperPure Beads (Roche, cat. no. 07983298001) in a two-step purification with a sample-to-reagent volume ratio of step 1—1:0.75 and step 2—1:0.85. Sequencing was performed on an Illumina NovaSeq 6000 instrument using the NovaSeq 6000 S1 Reagent Kit v1.5 (200 cycles) (Illumina, cat. no. 20028317) in a pair-end 2 × 100 cycle mode, using the standard procedure recommended by the manufacturer with a 0.5% addition of the PhiX control library (Illumina, cat. no. FC-110-3001).

### Genome assembly and data analysis

Sequence quality metrics for Illumina data were assessed using FASTQC version 0.12.0 (http://www.bioinformatics.babraham.ac.uk/projects/fastqc/) (21), and short reads were quality trimmed using fastp version 0.23.2 (22). Nanopore reads were quality filtered using NanoFilt version 2.8.0 (reads shorter than 1 kb and QV < 12 were discarded) (23). After residual adapter removal using Porechop version 0.2.4 (https://github.com/rrwick/Porechop), the data set was quality checked using NanoPlot version 1.41.6 (23).

Long-read assembly was performed using Trycycler version 0.5.3 pipeline (24). In brief, nanopore reads were initially assembled using four long read assemblers: flye v2.9, unicycler v0.4.8, Raven v1.8.1, and miniasm v0.3-r179. The long-read assemblies were then reconciled and circularized, and final consensus was generated followed by polishing with medaka (https://github.com/nanoporetech/medaka). Long-read assembled contigs were further polished using polypolish (https://github.com/rrwick/Polypolish) (24) and POLCA (25). All the possible sequence errors and misassemblies were further manually corrected using SeqMan software (DNAStar) to obtain a complete nucleotide sequence of the bacterial genome.

The assembled genomic sequences were annotated using Bakta (26). Plasmid replicon sequences were searched against the PLSDB database (https://ccb-microbe.cs.uni-saarland.de/plsdb/). ARGs were detected and localized in the genomic sequences using abricate (https://github.com/tseemann/abricate). MGEs were identified using mobileelementfinder (27), and the ARG-associated mobilome was characterized using VRprofile2 (28).

The plasmid sequences were visualized using the SnapGene Viewer software (SnapGene software from Dotmatics; available at snapgene.com). The linear plasmid sequences were compared using Easyfig software (29).

## RESULTS

### *E. coli* strain isolation

In total, 27 bacterial strains demonstrating growth indicative of *E. coli* were found using selective media; more specifically, 16 strains were isolated using EMB Agar and 11 via MacConkey Agar. Among the isolated bacteria, 12 were identified as *E. coli* through MALDI-TOF MS/MS analysis, of which four strains were isolated on EMB agar and eight on MacConkey agar. All bacterial strains were isolated on agar plates enriched with cefotaxime; however, no strains exhibited resistance to imipenem.

The antimicrobial susceptibility profiles of all strains were comprehensively determined utilizing the Vitek 2 Compact system (Data S1). Isolates that demonstrated analogous antimicrobial susceptibility profiles were aggregated, and their plasmid profiles were elucidated. Following the comparative analysis of plasmid profiles within these aggregates, two bacterial strains exhibiting potentially unique plasmid configurations were selected for total plasmid DNA sequencing.

### Genome sequencing and assembly

The genomes of the investigated strains were sequenced using a hybrid approach. Oxford Nanopore long-read technology (MINIon platform) was used to obtain as complete scaffolds as possible. The quality of the obtained sequences was then improved with Illumina short-read technology (2 × 100 nt in paired-end mode, NovaSeq 6000, approximately 100× coverage). The bacterial chromosome was assembled for both samples, resulting in an approximately 5 Mb contig. Four additional significant contigs were assembled for “kr1” and three for “kr2” (Table 1), and these were analyzed in detail.

**TABLE 1:** Plasmids identified in studied *E. coli* strains^*a*^

Strain no.	Origin	Plasmid no.	Plasmid name in our collection	Size (bp)
kr1	Cattle manure	1	pECmdr1.1	103,976
		2	pECmdr1.2	6,647
		3	pECmdr1.3	6,466
		4	pECmdr1.4	3,374
kr2	Cattle manure	1	pECmdr2.1	103,976
		2	pECmdr2.2	6,647
		3	pECmdr2.3	6,466

^*a*^ Those harboring ARGs are marked in gray.

### Plasmids

Seven plasmids belonging to two bacterial isolates were identified (Table 1). The complete genomes of *E. coli* are deposited as a part of “INART *Escherichia coli* genomes” bioproject (accession no. PRJNA942482). Two plasmids have more than 100,000 bp and may be classified as megaplasmids. These two identified plasmids harbored different ARGs and MGEs (Table 2). Plasmids that do not confer antibiotic resistance will be characterized and deposited further, as a part of an additional project.

**TABLE 2:** ARGs, MGEs, integron integrase genes, transposon transposase genes, conjugal transfer system genes, and heavy metal resistance genes^*a*^

Plasmid no.	Size (bp)	ARG	MGE	Integron integrase gene	Transposon transposase gene
^*b*^pECmdr1.1	103,976	*tet(A)* *dfrA14* *sul2* *aph(3″)-Ib* *aph (6)-Id* *bla*_TEM-1_ *bla*_CTX-M-15_ *qnrS1*	*cn_34182_IS5075* *cn_31036_IS26* *cn_3458_IS26* *cn_29961_IS26* *ISKpn19* *ISEc9* *IS5075* *IS26* *ISSen9*	*intI1*	*tnp*-IS3 *tnp-IS1* *tnp*-IS4321 *tnp*A *tnp*-IS1A *tnp*-IS91
pECmdr1.2	6,647	–^*c*^	–	–	–
pECmdr1.3	6,466	–	–	–	–
pECmdr1.4	3,374	–	–	–	–
^*b*^pECmdr2.1	103,976	*tet(A)* *dfrA14* *sul2* *aph(3″)-Ib* *aph (6)-Id* *bla*_TEM-1_ *bla*_CTX-M-15_ *qnrS1*	*cn_34182_IS5075* *cn_31036_IS26* *cn_3458_IS26* *cn_29961_IS26* *ISKpn19* *ISEc9* *IS5075* *IS26* *ISSen9*	*intI1*	*tnp*-IS3 *tnp*-IS1 *tnp*-IS4321 *tnp*A *tnp*-IS1A *tnp*-IS91
pECmdr2.2	6,647	–	–	–	–
pECmdr2.3	6,466	–	–	–	–

^*a*^ cn, composite transposons; IS, insertion sequences; and Tn, transposon.

^*b*^ Plasmids selected for analysis.

^*c*^ "–", not identified.

**Fig 1:**
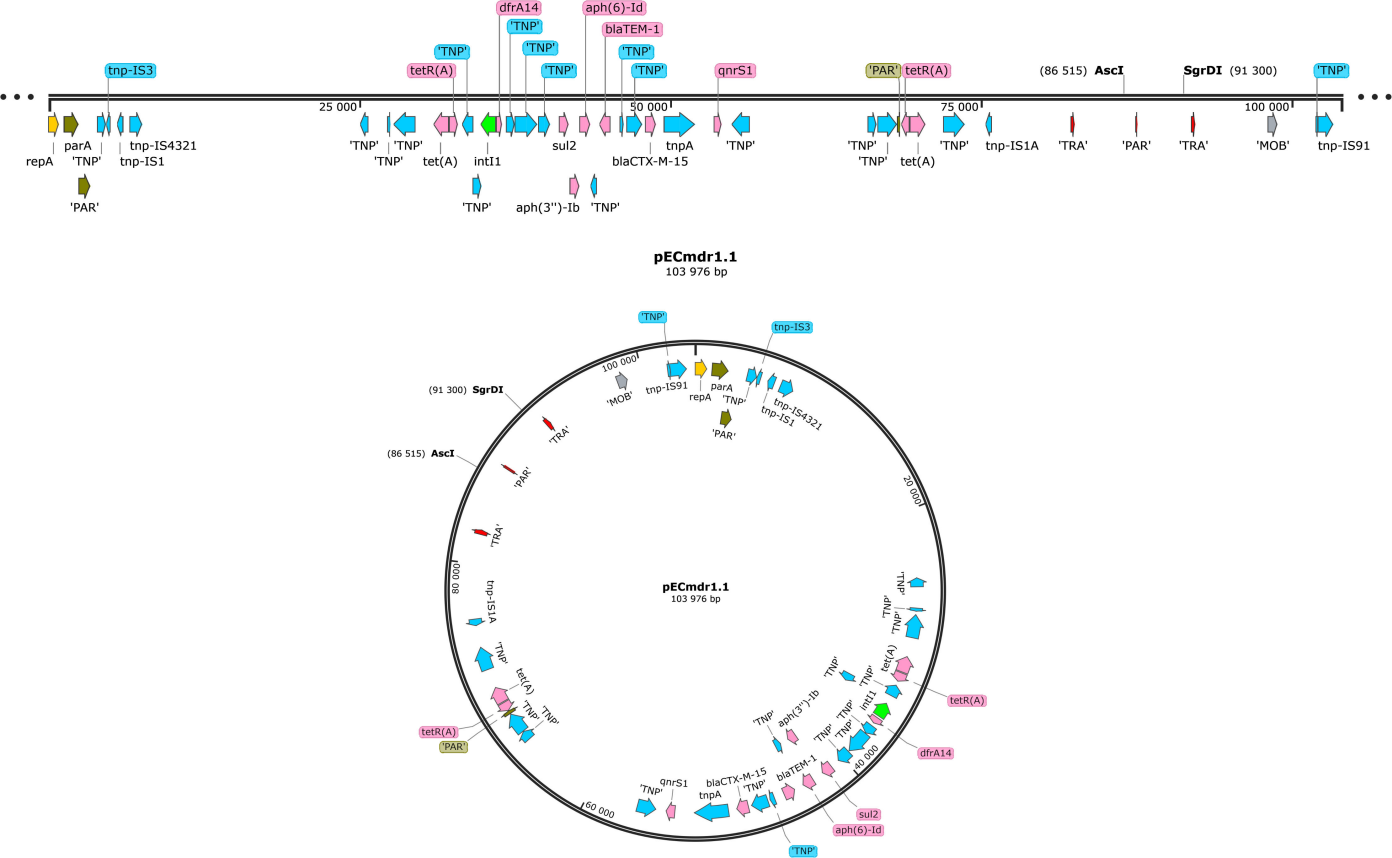
Linear and circular representation of pECmdr1.1 plasmid (pink—ARG, red—conjugal transfer genes TRA, blue—transposon transposase genes TNP, brown—heavy metal resistance genes, yellow—replication system REP, green—integron integrase, khaki—partitioning system PAR, gray—mob system MOB, plum—pilus system PIL; quotation marks indicate that the function of the gene has been assigned based on the homology of the protein it encodes).

**Fig 2:**
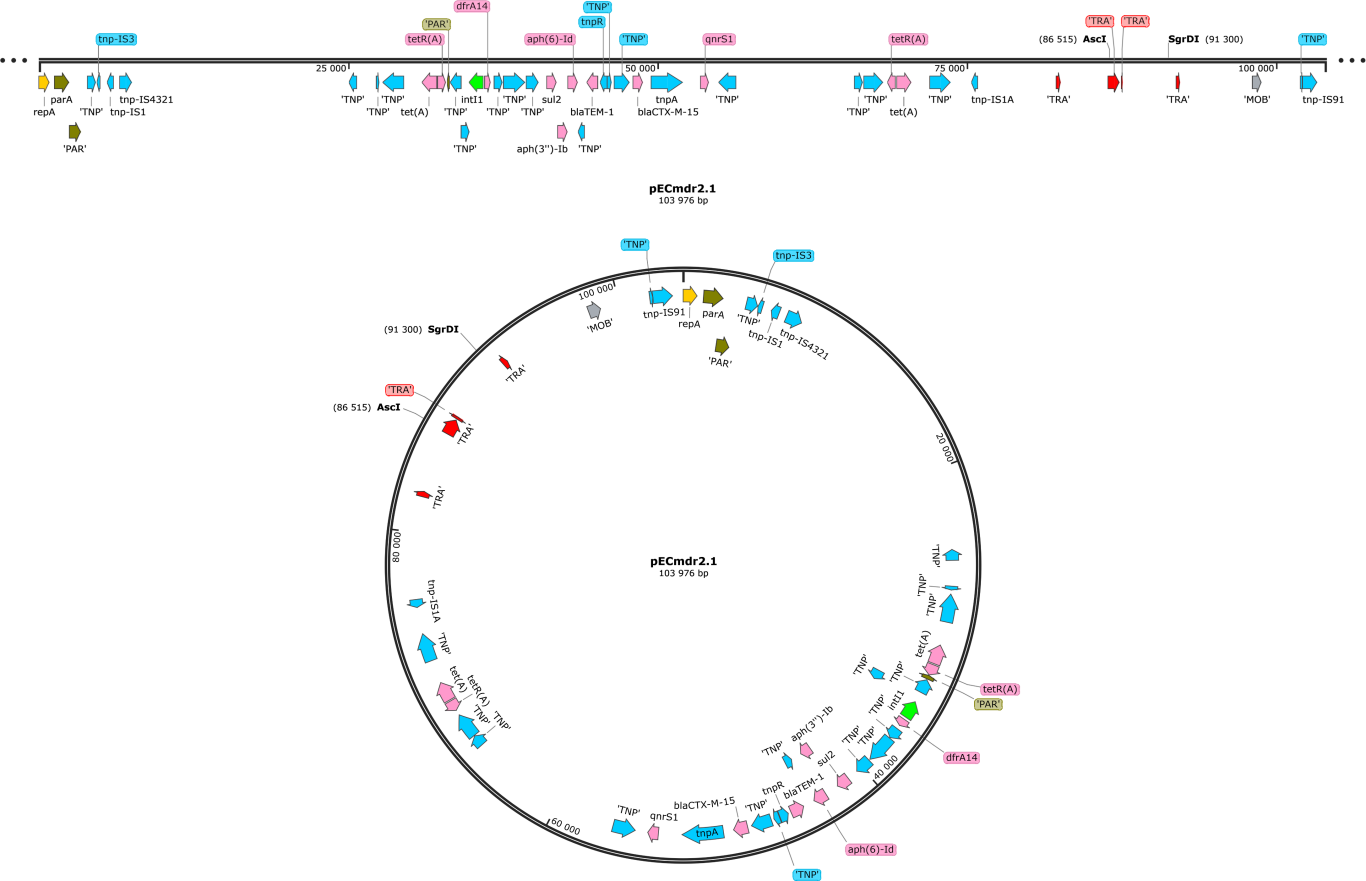
Linear representation of pECmdr2.1 plasmid (pink—ARG, red—conjugal transfer genes TRA, blue—transposon transposase genes TNP, brown—heavy metal resistance genes, yellow—replication system REP, green—integron integrase, khaki—partitioning system PAR, gray—mob system MOB, plum—pilus system PIL; quotation marks indicate that the function of the gene has been assigned based on the homology of the protein it encodes).

Figures 1 and 2 present the linear and circular plasmid structures of the bacteria, with particular regard to ARGs, MGEs, conjugal transfer genes (TRA), transposon transposase genes (TNF), heavy metal resistance genes, replication system (REP), integron integrase, and partitioning system elements (PAR).

**Fig 3:**
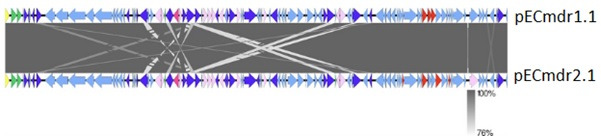
Comparison between pECmdr1.1 and pECmdr2.1 (linear map showing the genetic structure of circular plasmids) (yellow—replication system, red—conjugal transfer system, green—partitioning system, violet—ARG, blue—transposase genes, pink—integron integrase, and pale blue—other genes).

Although these plasmids are similar in size and carry genes, particularly ARG, MGE, PAR system, TNP system, TRA system, or REP system, they differ structurally (Fig. 3).

A detailed analysis of bacterial plasmid sequences deposited in the PLSDB plasmid database (https://ccb-microbe.cs.uni-saarland.de/plsdb/) (30, 31) found the plasmids identified during the study to be closely similar to previously deposited ones, albeit with some differences. The pECmdr1.1 plasmid has 39 similar hits, while pECmdr2.1 has 39 (identity threshold 0.99) [detailed data are collected as Data S2 (pECmdr1.1 ) and Data S3 (pECmdr1.2)]. Sequence analysis indicates that both plasmids belong to the incY incompatibility group. Neither plasmid has previously been reported in Poland, but they have been identified in many other countries. Plasmids similar to pECmdr1.1 and pECmdr2.1 have been found only in the soil, *Branta leucopsis* fecal sample (only in Finland), pig farm surroundings, and human clinical samples, not in cattle (Table 3).

**TABLE 3:** Region, isolation source, and host biosample of similar plasmids (plasmid identity 0.99)

Plasmid no.	Plasmid type	First reported	Region	Isolation source
pECmdr1.1	IncY_1 K02380	2002	France	Feces (*Branta leucopsis*)^a^
			China	
			USA	Feces (*Homo sapiens*)
			South Korea	Urine (*Homo sapiens*)
			Pakistan	Blood (*Homo sapiens*)
			Denmark	Wound (*Homo sapiens*)
			India	Bile (*Homo sapiens*)
			Mali	Sputum (*Homo sapiens*)
			Germany	
			Finland	Soil (environmental)^a^
			Portugal	Soil (pig farm floor)
			United Kingdom	
			Australia	
pECmdr2.1	IncY_1 K02380	2002	France	Feces (*Branta leucopsis*)^a^
			China	
			USA	Feces (*Homo sapiens*)
			South Korea	Urine (*Homo sapiens*)
			Pakistan	Blood (*Homo sapiens*)
			Denmark	Wound (*Homo sapiens*)
			India	Bile (*Homo sapiens*)
			Mali	Sputum (*Homo sapiens*)
			Germany	
			Finland	Soil (environmental)^a^
			Portugal	Soil (pig farm floor)
			United Kingdom	
			Australia	

^a^ Lack of more detailed data or data unpublished.

PLSDB data indicates that in addition to *E. coli*, similar plasmids have been found in other *Enterobacteriaceae*(Table 4).

**TABLE 4:** The host range for similar plasmids (plasmid identity 0.99)

Plasmid similar to	Identified in the following bacteria:
pECmdr1.1	*Enterobacter hormaechei* *Escherichia coli* *Escherichia fergusonii* *Klebsiella pneumoniae* *Salmonella enterica* subsp. *Enterica*
pECmdr2.1	*Enterobacter hormaechei* *Escherichia coli* *Escherichia fergusonii* *Klebsiella pneumoniae* *Salmonella enterica* subsp. *enterica*

**Fig 4:**
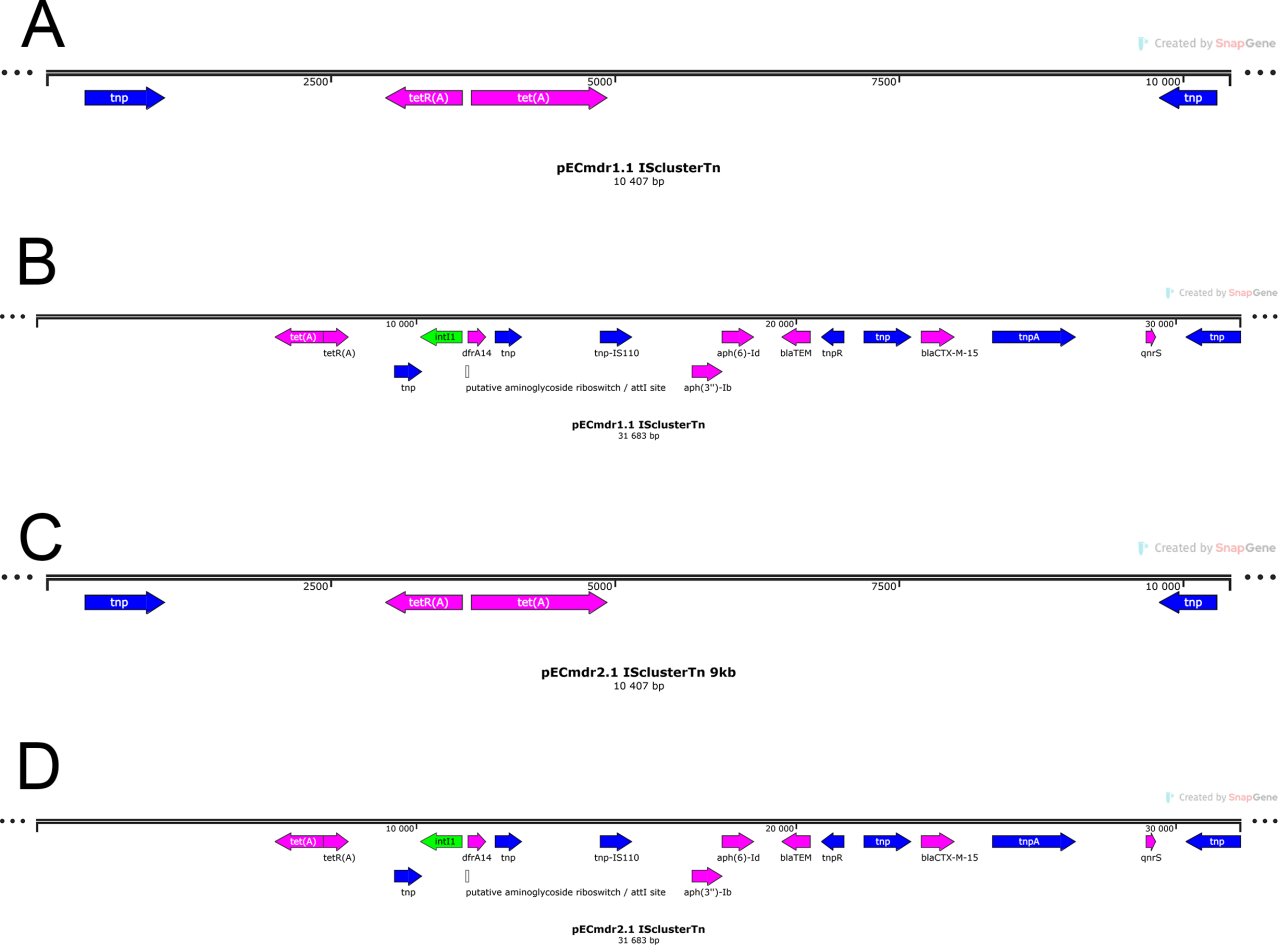
(A) pECmdr1.1 ISclusterTn (9,949 bp). (**B**) pECmdr1.1 ISclusterTn (31,306 bp). (**C**) pECmdr2.1 ISclusterTn (9,949 bp). (**D**) pECmdr2.1 ISclusterTn (31,306 bp).

By aligning the ARG-coding sequences with the defined and described MGE, it was possible to identify the ARGs located within the MGE, which were situated on the identified plasmids (Fig. 4A through D).

## DISCUSSION

The spread of antimicrobial resistance is recognized as a key global challenge to human health (32). *E. coli,* in particular, is known to cause many infections and is frequently associated with drug resistance, especially against cephalosporins (ESBL phenotype) (11). The risk related to infection caused by carbapenem-resistant, ESBL-producing *Enterobacteriaceae* has been highlighted by the World Health Organization as critical (15). Current research in microbiology suggests that the diversity of ARGs in plasmids can be linked to the varied environments where bacteria thrive. Studies have shown that certain ARGs, like *tet(A*), are commonly found in both clinical and environmental isolates (CARD) (33), indicating a widespread distribution and a potential for cross-environment transfer.

The high diversity of bacterial ARGs and the varied health risks associated with bacterial human pathogens require proper environmental risk assessment. ARGs from anthropogenic pollutants have considerable potential to influence environmental resistomes and be acquired by human pathogens; as such, they constitute a significant environmental and health threat. Human-animal-shared mobile ARGs have been identified in human, chicken, pig, and cattle guts, and studies suggest they may confer resistance to as many as six major antibiotic classes (tetracyclines, aminoglycosides, macrolide-lincosamide-streptogramin B, chloramphenicol, beta-lactams, and sulfonamides) at the same time. Additionally, there is a shared resistome between soil bacteria and human pathogens, and it is suggested that soil microbiota act as ARG reservoirs for human and animal pathogens (34, 35).

Our present findings identified two very similar plasmids harboring ARGs against at least six antimicrobial groups, making the identified bacterial strains multi-drug resistant (MDR). Both plasmids harbored the tetracycline resistance gene *tet(A)* (TetA is a tetracycline efflux pump found in many species of Gram-negative bacteria), the trimethoprim resistance gene *dfrA14* (dfrA14 is an integron-encoded dihydrofolate reductase found in Gram-negative bacteria), the sulfonamide resistance gene *sul2* (Sul2 is a sulfonamide-resistant dihydropteroate synthase of Gram-negative bacteria, usually found on small plasmids), the aminoglycoside resistance gene *aph(3″)-Ib* [APH(3″)-Ib is an aminoglycoside phosphotransferase encoded by plasmids, transposons, integrative conjugative elements, and chromosomes in *Enterobacteriaceae* and *Pseudomonas* spp.], the aminoglycoside resistance gene *aph(6)-Id* [APH(6)-Id is an aminoglycoside phosphotransferase encoded by plasmids, integrative conjugative elements, and chromosomal genomic islands in *Enterobacteriaceae*, *Providencia alcalifaciens*, *Pseudomonas* spp., *Vibrio cholerae*, *Edwardsiella tarda*, *Pasteurella multocida,* and *Aeromonas bestiarum*], *bla*_TEM-1_ (beta-lactam resistance gene; TEM-1 is a broad-spectrum beta-lactamase found in many Gram-negative bacteria; confers resistance to penicillins and first-generation cephalosporins), the beta-lactam resistance gene *bla*_CTX-M-15_ (CTX-M-15 is a beta-lactamase found in the *Enterobacteriaceae* family), and the quinolone-resistance gene *qnrS1* (QnrS1 is a plasmid-mediated quinolone resistance protein found in *Enterobacteriaceae*) (CARD) (33). The presence of all these resistance genes in *E. coli* from cattle manure is not unexpected since tetracyclines, beta-lactams, cephalosporins, aminoglycosides, trimethoprim, or quinolones are used to treat various infections in cattle, including respiratory diseases, mastitis, or dry cow therapy (36). The antibiotic use policy in veterinary medicine and animal husbandry adheres to the same regulations across the European Union. The use of antibiotic growth promoters is banned, as well as the prophylactic use of antibiotics. Any medical application must occur under the strict supervision of a veterinarian and be prescribed. However, according to 13th European Surveillance of Veterinary Antimicrobial Consumption (ESVAC) report, the quantity of antibiotics sold for food-producing animals in Poland ranks among the highest in Europe (second, after Cyprus; if measured in mg/PCU). The overall annual sales of veterinary antibiotics increased by 55.2% from 2011 to 2022 and 11.7% from 2021 to 2022. The most used are penicillin (69.1 mg/PCU; 35.3%), followed by tetracyclines (39.0 mg/PCU; 19.9%) and macrolides (28.8 mg/PCU; 14.7%). Consumption of first and second generation cephalosporins, as well as third and fourth, places Poland among the top 10 countries according to the 13th ESVAC report. According to the ESVAC report, the use of third and fourth generation cephalosporins in Poland has increased from 2010 to 2022 by 372.8 (37). As per the same report in Poland, most antibiotics are used in cattle farming, followed by poultry and pigs (1,480, 1,411, and 1,203 tonnes, respectively). During our study, we identified genes coding for resistance against the most applied antibiotic groups, as tetracyclines and penicillins, but not macrolides. We also found trimethoprim-, sulfonamide-, aminoglycoside-, and quinolones-resistance genes—antibiotics from these groups are being applied to food-producing animals in Poland but not so often than the first three (1.7, 8.9, 9.3, and 11.8 mg/PCU, respectively) (37). Our results prove that even strictly regulated antibiotic application policies in veterinary medicine may lead to antimicrobial resistance development.

Although the particular plasmids identified during the study have not been described previously, similar plasmids (threshold 0.99) have been reported both in countries with restrictive antimicrobial usage policies (e.g., Denmark) and in those with “flexible” ones (e.g., India and China). Resistance plasmids have been found across the globe (USA, Europe, and Asia), which implies that they are also widespread among people and the environment; indeed, they have been isolated from human clinical samples (urine and blood), soils, pig farm surroundings, and wild birds. No similar plasmids have previously been isolated in Poland, and none have originated from dairy cattle. The accumulation of so many ARGs on a single plasmid may result from the huge selection pressure placed on bacteria living in the intestines of dairy cattle due to the overuse of antibiotics in dairy cattle breeding (38).

Plasmid-encoded genes can arise from multiple sources and be further disseminated by HGT. Our present findings indicate the presence of beta-lactam-resistance genes originating in dairy cattle in various MGEs, such as insertional sequences or transposons (Fig. 4A through D). ESBL-producing *E. coli* is commonly isolated from community or hospital infections, human fecal carriage, and to an increasing degree from food-producing, companion, and wildlife animals, and the environment; however, particular plasmids similar to those found during this study have been found in the vast majority in clinical samples (39).

Our findings confirm previous observations that ESBL transmission is mainly driven by insertion sequences, transposons, integrons, and plasmids, which are homologous and may be isolated from both food-producing animals and humans (40). The widespread distribution of *E. coli* carrying the *bla*_CTX-M-15_ gene seems to be linked to a hot spot of insertion on the *bla*_TEM-1_-Tn2 transposon, which is largely found in broad host-range IncF plasmids (39); we found both of these genes to be located within transposon but on IncY plasmids. It has also been noted that plasmids expressing an ESBL phenotype also frequently carry genes encoding resistance to other commonly used antimicrobial drug classes, such as aminoglycosides, chloramphenicols, fluoroquinolones, or tetracyclines (40, 41); this was also noted during this study. Plasmid-mediated transfer of drug-resistance genes between various bacterial species is considered to be one of the most important mechanisms driving the spread of multidrug resistance (40).

Removing a factor responsible for selection pressure will result in the loss of a plasmid carrying genes that determine survival in unfavorable conditions; however, the plasmids can remain within the cell when it harbors more than one resistance determinant. Thus, even though some antibiotics were not applied during therapy, the bacteria can still carry identified plasmids due to cross-resistance (42).

The newly identified plasmids were included in the IncY incompatibility group, which, unlike IncN or IncP, is not a broad host-range incompatibility group. The prevalence of IncY plasmids seems to be limited to the *Enterobacteriaceae* family. Similar plasmids (threshold 0.99) have previously been identified in human clinical samples, wild animals, companion animals, livestock, poultry, and environmental samples, even in alfalfa sprouts (30, 31). In addition, IncY plasmids have been found in meat and other food samples. Balbuena-Alonso et al. (2) report that plasmids originating in food contaminated with *E. coli* may be transferred to pathogenic strains of *E. coli*.

Our findings have considerable clinical and environmental implications. The presence of MDR bacteria in various settings poses significant challenges to public health. Research emphasizes the need for robust antibiotic stewardship and environmental monitoring to manage the spread of resistance. The cost of treating a single infection caused by MDR bacteria is 165% higher than for non-MDR infection, with an incremental cost of 1,383 USD (43).

It is crucial that antibiotic-resistant bacterial strains and the plasmids coding for resistance are tracked effectively. Antibiotic resistance does not respect boundaries, and ARGs can move between bacterial strains in an almost uncontrolled manner. *E. coli* demonstrates considerable diversity and high adaptability and hence can inhabit the natural environment, animal intestines, and plants, where it can serve as both a harmless commensal and a dangerous pathogen. As such, this bacterium is an ideal vector for transmitting plasmids encoding drug resistance between different environments.

### Conclusion

The study characterizes two *E. coli* plasmids, each carrying ARGs and insertion elements, found in two representative isolates from dairy cattle manure. Such plasmids have not been previously reported in Poland, though similar ones have been recorded in crucial human pathogens in other countries. However, plasmids resembling pECmdr1.1 and pECmdr2.1 from cattle manure-derived *E. coli* have only been seen in natural environments and human clinical samples, not in farm animals.

The identified plasmids, isolated from *E. coli* obtained from cattle manure, confer resistance to beta-lactam antibiotics, including penicillins (even with beta-lactamase inhibitors like clavulanic acid), cephalosporins (second to fourth generation), aminoglycosides, fluoroquinolones, tetracycline derivatives (glycylcycline antibiotics), and chemotherapeutics/sulfonamides (trimethoprim/sulfamethoxazole). All identified resistance plasmids carry conjugal transfer genes, enabling HGT. The identified plasmids belong to the IncY group, which have been identified in *Enterobacteriaceae,* such as *E. coli*, *Salmonella* spp., and *Klebsiella pneumoniae*, though information on IncY plasmids remains limited.

## Data Availability

The genome sequences of *E. coli* strains generated during this study have been deposited in GenBank (NCBI) with the accession number PRJNA942482. Plasmid data sets generated during this study have been deposited in the University of Warsaw repository with the doi number https://doi.org/10.58132/YFTW53.

## SUPPLEMENTAL MATERIAL

Supplemental data 1 spectrum.00877-24-s0001.csv Isolated E. coli strains phenotypes.

Supplemental data 2 spectrum.00877-24-s0002.csv Plasmids similar to pECmdr1.1 characteristic.

Supplemental data 3 spectrum.00877-24-s0003.csv Plasmids similar to pECmdr2.1 characteristic.

ASM does not own the copyrights to Supplemental Material that may be linked to, or accessed through, an article. The authors have granted ASM a non-exclusive, world-wide license to publish the Supplemental Material files. Please contact the corresponding author directly for reuse.
